# X-ray and cryo-EM structures of inhibitor-bound cytochrome *bc*_1_ complexes for structure-based drug discovery

**DOI:** 10.1107/S2052252518001616

**Published:** 2018-02-20

**Authors:** Kangsa Amporndanai, Rachel M. Johnson, Paul M. O’Neill, Colin W. G. Fishwick, Alexander H. Jamson, Shaun Rawson, Stephen P. Muench, S. Samar Hasnain, Svetlana V. Antonyuk

**Affiliations:** a Molecular Biophysics Group, Institute of Integrative Biology, Faculty of Health and Life Sciences, University of Liverpool, Liverpool L69 7ZB, England; b School of Biomedical Sciences, Faculty of Biological Sciences, University of Leeds, Leeds LS2 9JT, England; c Astbury Centre for Structural and Molecular Biology, University of Leeds, Leeds LS2 9JT, England; d School of Chemistry, University of Leeds, Leeds LS2 9JT, England; e Department of Chemistry, University of Liverpool, Liverpool L69 7ZD, England

**Keywords:** cryo-electron microscopy, cryo-EM, membrane proteins, electron-transport chain, malaria, cytochrome *bc*_1_

## Abstract

Cytochrome *bc*_1_, a dimeric multi-subunit electron-transport protein embedded in the inner mitochondrial membrane, is a major drug target for the treatment and prevention of malaria and toxoplasmosis. Structural studies of cytochrome *bc*_1_ from mammalian homologues co-crystallized with lead compounds have underpinned structure-based drug design to develop compounds with higher potency and selectivity. However, owing to the limited amount of cytochrome *bc*_1_ that may be available from parasites, all efforts have been focused on homologous cytochrome *bc*_1_ complexes from mammalian species, which has resulted in the failure of some drug candidates owing to toxicity in the host. Crystallographic studies of the native parasite proteins are not feasible owing to limited availability of the proteins. Here, it is demonstrated that cytochrome *bc*_1_ is highly amenable to single-particle cryo-EM (which uses significantly less protein) by solving the apo and two inhibitor-bound structures to ∼4.1 Å resolution, revealing clear inhibitor density at the binding site. Therefore, cryo-EM is proposed as a viable alternative method for structure-based drug discovery using both host and parasite enzymes.

## Abstract 2

Structures of inhibitor-bound bovine cytochrome *bc*_1_ were determined by cryo-EM and compared with X-ray crystallographic structures, demonstrating that cryo-EM could be a feasible tool for structure-based drug discovery.

## 1. Introduction   

Cytochrome *bc*_1_ (*bc*_1_) is an established drug target against apicomplexan parasites, for example *Plasmodium falciparum* (Nixon *et al.*, 2013 ▸) and *Toxoplasma gondii* (Doggett *et al.*, 2012 ▸), which are the causative agents of malaria and toxoplasmosis, respectively. According to data from the World Health Organization, the malaria infection rate has declined by 41% since the turn of the century, but 212 million cases still occurred globally in 2015 (World Health Organization, 2016 ▸). *T. gondii* is involved in ten million cases of foodborne illness annually. Moreover, *T. gondii* causes congenital toxoplasmosis in ∼190 000 infants per year following congenital transmission (Torgerson & Mastroiacovo, 2013 ▸). *bc*_1_, which exists as a multi-subunit heterodimer embedded in the inner mitochondrial membrane, is a validated drug target for both of these apicomplexan parasites (Schägger *et al.*, 1986 ▸). The subunit composition of the complex can vary between species. For instance, there are three subunits in bacteria (Berry *et al.*, 2004 ▸), ten subunits in yeast (Hunte *et al.*, 2000 ▸) and 11 subunits in vertebrates (Xia *et al.*, 1997 ▸; Zhang *et al.*, 1998 ▸). However, all *bc*_1_ complexes have a catalytic core containing three essential subunits: cytochrome *b*, cytochrome *c*_1_ and the Rieske iron–sulfur protein (Yang & Trumpower, 1986 ▸). *bc*_1_ is the central component of the mitochondrial electron-transfer chain which is responsible for the electron flow from ubiquinol to cytochrome *c*. This occurs *via* the oxidation of ubiquinol and the reduction of ubiquinone in a process called the Q cycle (Mitchell, 1976 ▸). The Q cycle takes place in the Q_i_ and Q_o_ binding pockets within cytochrome *b* (Erecińska *et al.*, 1972 ▸). Ubiquinol is oxidized to ubiquinone in the oxidative (Q_o_) site, with two protons released into the intermembrane space, while ubiquinone binds to the reductive (Q_i_) site, where it is reduced to ubiquinol and takes two protons from the matrix (Mitchell, 1976 ▸). This process allows proton translocation from the matrix space to the intermembrane space, thus increasing the electrochemical gradient across the membrane.

The first *bc*_1_ structure from bovine mitochondria was determined by X-ray crystallography at 3.0 Å resolution in 1997 (Xia *et al.*, 1997 ▸). Since then, numerous crystal structures of *bc*_1_ from various species have been reported, some with improved resolution, for example bovine (2.1 Å; Huang *et al.*, 2005 ▸), chicken (2.7 Å; Hao *et al.*, 2012 ▸), yeast (1.9 Å; Solmaz & Hunte, 2008 ▸) and *Rhodobacter* (2.4 Å; Esser *et al.*, 2008 ▸), thus providing a more detailed understanding of the enzyme mechanism and structure–function relationship.

In apicomplexan parasites, oxidized ubiquinone from *bc*_1_ is used by dihydroorotate dehydrogenase (DHODH) to generate orotate, which is an essential intermediate in pyrimidine biosynthesis (Painter *et al.*, 2007 ▸). Therefore, inhibition of *bc*_1_ leads to a collapse in pyrimidine production, causing parasite death (Srivastava & Vaidya, 1999 ▸). Atovaquone is an analogue of ubiquinone that shows the ability to act as a competitive inhibitor at the Q_o_ site of cytochrome *bc*_1_ (Fry & Pudney, 1992 ▸). It has been widely used in combination with proguanil (known as Malarone) for the treatment of uncomplicated malaria and its prevention (Shanks *et al.*, 1998 ▸), and atovaquone can also be used to treat toxoplasmosis (Pearson *et al.*, 1999 ▸). Point mutations within the Q_o_ site have been found in atovaquone-resistant strains of malaria (Srivastava *et al.*, 1999 ▸) and toxoplasmosis (McFadden *et al.*, 2000 ▸) parasites, and structural analysis confirmed that these mutations significantly reduce the antiparasite activity of atovaquone by preventing it from binding to the Q_o_ pocket (Birth *et al.*, 2014 ▸). Owing to the emergence of drug-resistant malaria, new compounds with improved potency and pharmacokinetic properties are urgently required for the eradication of malaria. Based on atovaquone, a number of lead compounds, such as 4(1*H*)-pyridones (Bueno *et al.*, 2012 ▸) and 4(1*H*)-quinolones (Biagini *et al.*, 2012 ▸), have been designed to target the Q_o_ site of apicomplexan parasites (Supplementary Fig. S1). GSK932121 is a 4(1*H*)-pyridone lead compound that has been found to be highly active against atovaquone-resistant *P. falciparum* (Bueno *et al.*, 2012 ▸), but failed in a first human trial because of acute cardiotoxicity (Jiménez-Díaz *et al.*, 2009 ▸). The root of this issue was exposed by the crystal structure, which showed the unexpected binding of GSK932121 to the Q_i_ site of bovine *bc*_1_ (Capper *et al.*, 2015 ▸). 2-Pyridyl­quinolones have been developed for antimalarial activity, with a mechanism of dual inhibition of NADH:ubiquinone oxidoreductase (*Pf*NDH2) and *bc*_1_ (Pidathala *et al.*, 2012 ▸; Biagini *et al.*, 2012 ▸). One quinolone, SCR0911, has been reported to have improved solubility and metabolic stability, with nanomolar activity against *P. falciparum* (Charoen­sutthivarakul *et al.*, 2015 ▸).

Structure-based drug-design (SBDD) programmes are reliant upon high-resolution structures of the target protein being solved (Renaud *et al.*, 2016 ▸; Anderson, 2003 ▸). When complexed with their natural substrate or an inhibitor molecule, they can provide essential information for the design of new compounds which are highly selective for their target. Previously, this has been underpinned by X-ray crystallo­graphy; however, owing to the recent advances in single-particle cryo-electron microscopy (cryo-EM), an increasing number of high-resolution structures have been determined, therefore the technique has the potential to play a role in SBDD programmes (Rawson *et al.*, 2017 ▸). For example, a 2.3 Å resolution cryo-EM structure of inhibitor-bound human p97 ATPase identified an allosteric inhibition mechanism that enables a structural basis for cancer drug design (Banerjee *et al.*, 2016 ▸). Cryo-EM is particularly useful for more challenging targets such as large macromolecular complexes, viruses and membrane proteins, as illustrated by the Fab–RV-B14 complex (2.26 Å resolution; Dong *et al.*, 2017 ▸) and the membrane protein TRPV1 (2.9 Å resolution; Gao *et al.*, 2016 ▸). Membrane proteins remain challenging to study using X-ray crystallo­graphy as they face hurdles in overexpression, the quantity of highly purified protein produced and the quality of the crystals obtained (Carpenter *et al.*, 2008 ▸). By requiring less sample for structural characterization (micrograms instead of milligrams), cryo-EM can overcome these issues (Rawson *et al.*, 2016 ▸). Moreover, the structure of the mammalian mitochondrial respirasome supercomplex has recently been determined to an overall resolution of 4.0 Å, which highlights how the individual components in the electron-transport chain interact with one another, which had not previously been seen using X-ray crystallography (Wu *et al.*, 2016 ▸; Guo *et al.*, 2017 ▸).

Here, we present a novel, co-crystal structure of SCR0911-bound bovine *bc*_1_ (at 3.1 Å resolution) and compare it with a previously published crystal structure of the bovine *bc*_1_–GSK932121 complex to discuss the differences in binding of these two different families of inhibitors. Moreover, we then show how cryo-EM has the potential to obtain structures at a similar resolution at which it is possible to see strong inhibitor density. By determining the cryo-EM structures of bovine apo *bc*_1_, *bc*_1_–GSK932121 and *bc*_1_–SCR0911 to 4.1 and 4.4 Å resolution, we can, for the first time, compare the information obtained by cryo-EM and X-ray crystallography. The present study demonstrates that cryo-EM could allow us to study the *bc*_1_ complex in systems where the production of sufficient quantities of native protein may be severely limiting for crystallization.

## 2. Methods   

### 2.1. Preparation of *bc*_1_   

*bc*_1_ was purified as described previously (Smith, 1967 ▸). Briefly, bovine mitochondria were isolated from fresh bovine hearts. The mitochondrial protein was quantified by the bicinchoninic acid (BCA) assay (Smith *et al.*, 1985 ▸) and solubilized in 50 m*M* potassium phosphate pH 7.5, 250 m*M* NaCl, 0.5 m*M* EDTA, 0.1 m*M* phenylmethylsulfonyl fluoride with the addition of 1 mg dodecylmaltoside (DDM) per 1 mg of mitochondrial protein. The suspension was centrifuged at 200 000*g* for 1 h at 4°C. The supernatant was loaded onto a 50 ml DEAE Sepharose CL6B (GE Healthcare) column pre-equilibrated in and washed with three column volumes of 50 m*M* potassium phosphate pH 7.5, 250 m*M* NaCl, 0.5 m*M* EDTA, 0.01% DDM and then eluted using a linear gradient from 250 to 500 m*M* NaCl. *bc*_1_ fractions were pooled and concentrated in a centrifugal ultrafilter (100 kDa molecular-weight cutoff). The protein was loaded onto a 120 ml Sephacryl S300 column (GE Healthcare) equilibrated in 20 m*M* K-MOPS pH 7.2, 100 m*M* NaCl, 0.5 m*M* EDTA, 0.01% DDM and eluted at a flow rate of 0.5 ml min^−1^. *bc*_1_ fractions were pooled and concentrated to a concentration of 40 mg ml^−1^. Increasing amounts of PEG 4000 were added stepwise to precipitate *bc*_1_. The protein started precipitating at 2% PEG 4000, and pure cytochrome *bc*_1_ fractions were obtained between 2.5 and 4% PEG 4000. The *bc*_1_ pellet was resolubil­ized in 25 m*M* potassium phosphate pH 7.5, 100 m*M* NaCl, 0.5 m*M* EDTA, 0.015% DDM and then buffer-exchanged in a centrifugal ultrafilter to remove residual PEG before being adjusted to 5 µ*M*. 50 m*M* inhibitor stock solution in DMSO was added to a tenfold molar excess and incubated at 4°C overnight. The specific activity of cytochrome *c* reduction was measured at 23°C as described in Supplementary Table S1. The specific activity of the purified bovine *bc*_1_ was 10.9 µmol min^−1^ n*M* ^−1^ at 23°C.

### 2.2. X-ray crystallography   

The inhibitor-bound *bc*_1_ was mixed with 1.6% 6-*O*-(*N*-heptylcarbamoyl)-methyl-β-d-glucopyranoside (HECAMEG) and concentrated to 40 mg ml^−1^. The protein solution was mixed with an equal volume of reservoir solution (50 m*M* potassium phosphate pH 6.8, 100 m*M* NaCl, 3 m*M* NaN_3_, 10–13% PEG 4000). Crystals were grown by the hanging-drop method at 4°C. Bipyramidal red crystals appeared amongst the precipitate after 2–3 d. Single crystals were harvested in nylon loops, soaked stepwise in 50% ethylene glycol and 50 m*M* potassium phosphate pH 6.8, 100 m*M* NaCl, 3 m*M* NaN_3_, 10–13% PEG 4000 and were then flash-cooled in liquid nitrogen. The cooled crystals were tested for diffraction at 100 K using a home X-ray source at Barkla X-ray Laboratory, University of Liverpool. The best diffracting crystals were sent to the I03 beamline at Diamond Light Source, UK. A single data set was collected with a PILATUS3 6M detector from a crystal at 100 K using a wavelength of 0.98 Å. Data were processed using *iMosflm* (Battye *et al.*, 2011 ▸) and scaled using *AIMLESS* (Evans & Murshudov, 2013 ▸). The structure was solved by molecular replacement with *MOLREP* (Vagin & Teplyakov, 2010 ▸) using the *bc*_1_ structure with PDB code 4d6u (Capper *et al.*, 2015 ▸) as the starting model; cofactors and ligands were removed from the starting model. Jelly-body refinement was carried out with *REFMAC*5 (Murshudov *et al.*, 2011 ▸) using the *ProSMART* option (Nicholls *et al.*, 2014 ▸) to assist low-resolution refinement during the first cycles of the refinement. TLS parameters for each of ten chains were introduced and refined at the final stages of the refinement to reflect the different levels of flexibility of the chains. The model was manually rebuilt in *Coot* (Emsley & Cowtan, 2004 ▸) between refinement cycles. In addition to protein residues and the inhibitor molecule, lipids (cardiolipin and 1,2-dimyristoyl-*sn*-glycero-3-phosphocholine and 1,2-dihexanoyl-*sn*-glycero-3-phosphoethanolamine) were added to the model, along with DDM detergent, short fragments of PEG molecules and the phosphate groups of less defined lipids. The inhibitor molecules were produced by using *JLigand* (Lebedev *et al.*, 2012 ▸). Data collection and refinement statistics are shown in Supplementary Table S2.

### 2.3. Electron microscopy   

Purified bovine cytochrome *bc*_1_ was buffer-exchanged into 25 m*M* Tris pH 7.5, 100 m*M* NaCl, 0.5 m*M* EDTA and 0.015% DDM or 0.01% lauryl maltose neopentyl glycol (LMNG) by several dilutions in a centrifugal ultrafilter and adjusted to a concentration of 5 mg ml^−1^. 3 µl aliquots of the sample were applied onto Quantifoil Cu R1.2/1.3 holey carbon grids, which had been glow-discharged for 30 s using a Pelco glow-discharge unit. An FEI Vitribot was used to blot the grids for 6 s (blot force 6) at 100% humidity and 4°C before plunging them into liquid ethane. The grids were loaded into an FEI Titan Krios transmission electron microscope (Astbury Biostructure Laboratory, University of Leeds) operating at 300 kV. Data were collected on a Falcon III direct electron detector operated in integrating mode. Automated data collection was performed using the *EPU* software, a defocus range of −1 to −4 µm and a magnification of 75 000×, which yielded a pixel size of 1.065 Å for the two inhibitor-bound data sets, which were collected with slightly different dose rates: *bc*_1_–GSK932121 had a total dose of 75 e^−^ Å^−1^ over a 1.5 s exposure, whereas *bc*_1_–SCR0911 had a total dose of 85 e^−^ Å^−1^ over a 2 s exposure. The apo *bc*_1_ data set was collected on a Titan Krios operating at 300 kV at the Astbury Biostructure Laboratory, which was fitted with a Gatan K2 electron detector. Automated data collection was carried out using the *EPU* software, a defocus range of −1 to −4 µm and a magnification of 75 000×, which yielded a pixel size of 1.047 Å. The total dose was 44 e^−^ Å^−1^ over a 12 s exposure, which was split into 20 frames (Supplementary Table S3). The apo, GSK932121-bound and SCR0911-bound *bc*_1_ data sets were collected over 3 d, resulting in 3256, 8840 and 7893 micrographs, respectively.

### 2.4. Image processing   

All image processing was performed in *RELION* 2.0 (Scheres, 2012 ▸) unless otherwise stated. For all data sets the initial drift and CTF correction was carried out using *MotionCor*2 (Zheng *et al.*, 2017 ▸) and *Gctf* (Zhang, 2016 ▸), respectively. For each data set, an initial subset of ∼2000 particles were manually picked and extracted into a 200 × 200 pixel box. These particles underwent two-dimensional classification to generate two-dimensional references to facilitate auto-picking. The total numbers of particles picked for GSK932121-bound, SCR0911-bound and apo *bc*_1_ were 466 865, 629 258 and 260 201, respectively, with the apo data set having the smallest number of particles owing to it having the fewest number of micrographs. For each data set, the particles underwent an initial round of two-dimensional classification, with those classes that displayed clear secondary-structure detail being taken forward to three-dimensional classification and split into three classes. Two of the three classes generated a high-quality *bc*_1_ reconstruction with clearly visible secondary-structure information. The particles from these two classes were recombined to form the final data sets. For the apo *bc*_1_ data set 57 571 particles were three-dimensionally refined using *C*2 symmetry to 4.4 Å resolution. For the SCR0911 (114 130 particles) and GSK932121 (232 910 particles) inhibitor-bound data sets, a global resolution of 4.1 Å was obtained for each map. Further rounds of two- and three-dimensional classification were performed on all of the data sets, including using soft masks around different regions, including the Rieske domain and the transmembrane region, but the resolution and map quality did not improve. The starting model for the reconstructions was a crystal structure low-pass filtered to 60 Å. These models were also independently generated *ab initio* in the absence of a starting model using both *RELION* and *cryo­SPARC*, resulting in an in­distinguishable map from that seeded with a low-pass filtered starting model (Scheres, 2012 ▸; Punjani *et al.*, 2017 ▸). Existing crystal structures were rigidly fitted into the maps using *UCSF Chimera* (Pettersen *et al.*, 2004 ▸) before *MDFF* (Trabuco *et al.*, 2010 ▸) was used to flexibly fit the model into the map. The inhibitor-bound models underwent model relaxation in *Rosetta* before being refined using *PHENIX* (Adams *et al.*, 2010 ▸). The maps were then inspected manually in *Coot* (Emsley & Cowtan, 2004 ▸) and the model was corrected for any errors in refinement and the placement of residues (Supplementary Table S3). All figures were produced using *UCSF Chimera* (Pettersen *et al.*, 2004 ▸) or *PyMOL* (Schrödinger).

### 2.5. Chemistry   

The synthesis of SCR0911 was carried out as described by Charoensutthivarakul *et al.* (2015 ▸).

## 3. Results   

### 3.1. Crystal structure of inhibitor-bound *bc*_1_   

**Figure 1:**
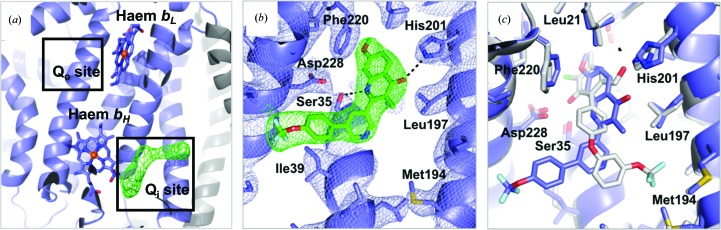
Crystal structure of the Q_i_ site of bovine *bc*_1_ with the SCR0911 inhibitor bound. (*a*) Cartoon representation of cytochrome *b* (blue) and the Rieske protein (grey). The Q_o_ and Q_i_ sites of the cytochrome *b* subunit are highlighted in black boxes. Haems *b*_L_ and *b*_H_ are shown as blue sticks. Omit *F*_o_ − *F*_c_ electron density (in green) is contoured at the 3σ level, showing density for the SCR0911 inhibitor. (*b*) SCR0911 inhibitor bound in the Q_i_ site. Residues and inhibitor are shown as sticks. The 2*F*_o_ − *F*_c_ electron-density map is contoured at the 1σ level, with the inhibitor density coloured green and the protein density in purple. Hydrogen bonds between inhibitors and residues are illustrated by black dashed lines. (*c*) The crystal structures of the Q_i_ site in *bc*_1_–SCR0911 (purple) and *bc*_1_–GSK932121 (grey) have been superimposed, showing the different binding modes of the pyridone (GSK932121, grey) and quinolone (SCR0911, purple) inhibitors.

The quinolone SCR0911 was co-crystallized with bovine *bc*_1_ in space group *P*6_5_22 and data were collected to 3.1 Å resolution. The resulting structure showed clear and continuous density for most of the protein. The 6.4 kDa subunit 11 could not be resolved within the structure; it may be lost during purification or be too mobile to be resolved. *bc*_1_ structures have been reported that show subunit 11 (Xia *et al.*, 1997 ▸; Esser *et al.*, 2004 ▸) and these were purified using ammonium sulfate precipitation protocols (Yu *et al.*, 1974 ▸). For the structures reported here, the protein was purified by DEAE ion-exchange chromatography followed by gel filtration (Iwata *et al.*, 1998 ▸) with the addition of a PEG fractionation step. Sub­units 1, 2, 7, cytochrome *b* and cytochrome *c*_1_ (chains *A*, *B*, *F*, *C* and *D*, respectively) are better defined in our structure, while the Rieske protein has weaker electron density and high temperature factors (the *B* factors for chains *E* and *I* are given in Supplementary Table S2). Chain *I*, which corresponds to the truncated N-terminal part of the Rieske protein, lacks the first 32 amino acids and adopts a conformation closer to that defined in the stigmatellin- and antimycin-bound *bc*_1_ structure (PDB entry 1ppj; Huang *et al.*, 2005 ▸). No additional density is seen within the Q_o_ site, suggesting that the quinolone solely inhibits the Q_i_ site (Fig. 1 ▸ *a*). There is additional density near haem *b*_H_ in the Q_i_ site which is not accommodated by the protein, and its size and shape allows unambiguous placement of the quinolone compound SCR0911. After refinement, the compound fits well into the 2*F*_o_ − *F*_c_ electron-density map (Fig. 1 ▸ *b*), and its temperature factors of ∼75 Å^2^ are consistent with those of the surrounding residues and are slightly higher than those of the haem *b*_H_ atoms (∼65 Å^2^). The carbonyl and amine groups of the quinolone head group are placed close to His201 and Ser35 (at distances of 3.1 and 3.2 Å, respectively), allowing hydrogen-bond formation. The bicyclic tail of SCR0911 is placed away from haem *b*_H_ towards the hydrophobic residue Ile39. Crystal structure alignment of SCR0911-bound *bc*_1_ with the previously determined structure of GSK932121-bound *bc*_1_ (PDB entry 4d6u; Capper *et al.*, 2015 ▸) illustrates that both the pyridone and quinolone compounds selectively inhibit the Q_i_ site but adopt different binding modes (Fig. 1 ▸ *c*). There are minor conformation changes in the Q_i_-site residues affected by the inhibitors. The pyridone head of GSK932121 is flipped over, forming hydrogen bonds between the carbonyl group and Ser35 and between the 2-hydroxymethyl substituent and His201 (Fig. 1 ▸ *c*). For SCR0911, the head group of the quinolone forms two hydrogen bonds to the carbonyl group of His201 and the amine group of Ser35. In other bovine *bc*_1_ crystal structures, His201, Ser35 and Asp228 make strong interactions with ubiquinol (Gao *et al.*, 2003 ▸) and Q_i_ inhibitors, including antimycin (Huang *et al.*, 2005 ▸) and ascochlorin (Berry *et al.*, 2010 ▸). The hydrogen bonds to Ser35 and His201 contribute to the ability of SCR0911 and GSK932121 to tightly occupy the Q_i_ site and prevent the native substrate, ubiquinone, binding in the active site. The different binding interactions of the head group result in the diaryl ether tail of GSK932121 packing in a hydrophobic cavity defined by Met190 and Met194. The bicyclic tail of SCR0911 is packed in the hydrophobic pocket formed by Gly38 and Ile39, with its trifluoromethyl group directed towards Ala232 (Fig. 1 ▸ *c*). These different binding modes of the pyridone and quinolone inhibitors might influence the binding affinity to the bovine enzyme. GSK932121 and SCR0911 were investigated for off-target inhibition of bovine *bc*_1_ (Supplementary Table S1). Single-point *bc*_1_ inhibition assays showed that SCR0911 has significantly decreased binding for mammalian target than GSK932121 at 100 n*M*: 9% inhibition compared with 64%. The binding behaviour of SCR0911 in the bovine Q_i_ site could suggest a direction for the design of future lead compounds with low affinity for mammalian *bc*_1_.

Although bovine and human cytochrome *b* show ∼80% conservation, the conservation between human and *P. falciparum* is only 40%. Surprisingly, the Q_o_ site is the most conserved region, with 65% conservation. The N-terminus of the parasite cytochrome *b*, which constitutes half of the Q_i_ site, is four residues shorter and has 39% conservation in comparison with mammalian homologues, leading to considerable differences in the Q_i_ binding site (Supplementary Fig. S2), which may offer a way to rationally optimize lead compounds to give more selective inhibitors of the parasite enzyme. In the case of GSK932121, Met190 and Met194 are replaced by Leu and Phe, respectively, in *P. falciparum* *bc*_1_, which could be beneficial for the design of pyridone derivatives. Interestingly, Ser35, which forms a hydrogen bond to the quinolone molecule, is replaced by a bulky Phe residue that would form a steric clash with SCR0911 in the parasite enzyme if the binding modes were the same. Therefore, to bind within *P. falciparum* *bc*_1_ SCR0911 must adopt a different binding mode in the parasite enzyme to the mode observed in the bovine crystal structure. Therefore, it is challenging to work on improving the selectivity of the inhibitor without the experimental structure of parasite *bc*_1_.

### 3.2. Structures of apo and inhibitor-bound *bc*_1_ from cryo-EM   

**Figure 2:**
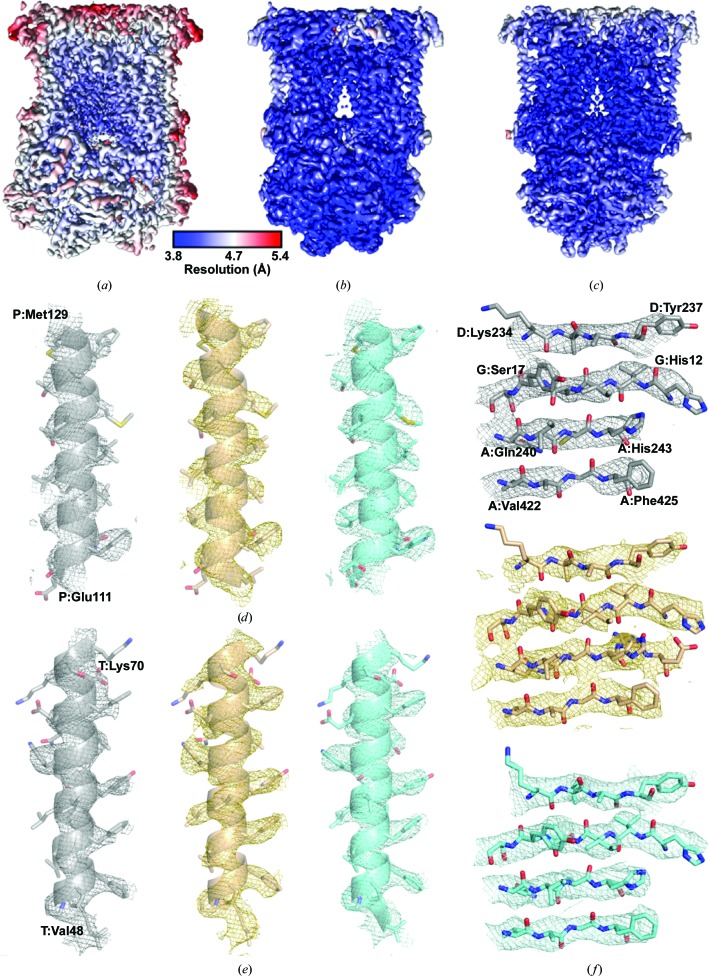
The bovine *bc*_1_ structures determined by cryo-EM. Local resolution maps, coloured on the same scale, of (*a*) apo *bc*_1_, (*b*) *bc*_1_–GSK932121 and (*c*) *bc*_1_–SCR0911, which show that the core of the complex is at the highest resolution and that the Rieske protein is the poorest resolved feature in the map. A comparison of the map quality within each map (coloured grey, gold and cyan for the apo, GSK932121-bound and SCR0911-bound structures, respectively) is shown for two representative transmembrane helices (*d*, *e*) and a β-strand within the soluble domain (*f*). In (*d*)–(*f*), the density was contoured at 4σ, with the side chains being better resolved in the two inhibitor-bound structures.

We used cryo-EM to study the structure of *bc*_1_ in complex with inhibitors from two different families with a pyridone (GSK932121; Bueno *et al.*, 2012 ▸) or quinolone (SCR0911; Charoensutthivarakul *et al.*, 2015 ▸) core, along with the apo complex. The apo data set was resolved to a global resolution of 4.4 Å and the two inhibitor-bound complexes were both determined to 4.1 Å resolution as calculated by Fourier shell correlation (FSC = 0.143; Scheres & Chen, 2012 ▸; Supplementary Fig. S2). For all three maps the local resolution was calculated using *RELION* (Figs. 2 ▸ *a*, 2 ▸ *b* and 2 ▸ *c*), showing that the core of the complex was resolved to a higher resolution than the global average (4.2 Å for the apo *bc*_1_ map and 3.8–4.0 Å for the inhibitor-bound maps). Moreover, the poorest resolved region in each map was consistently the Rieske protein, which was at ∼6 Å resolution in all maps. The iron–sulfur cluster within this region is responsible for bifurcated electron transfer from oxidation of ubiquinol in the Q_o_ site to cytochrome *c*_1_ in a *bc*_1_ catalytic cycle. Previously, it had been reported that this domain is mobile and can exist in different conformations (Iwata *et al.*, 1998 ▸; Gao *et al.*, 2003 ▸; Esser *et al.*, 2004 ▸, 2006 ▸). The other haem groups in the cryo-EM structure of *bc*_1_ are the strongest features within the map. However, our inability to resolve the iron–sulfur cluster, which should also be a strong feature, leads us to conclude that it is mobile and adopts different positions. Attempts to separate different conformational states of the iron–sulfur cluster using soft masks around the region were unsuccessful. This could suggest that rather than being in distinct conformations, the Rieske protein is more flexible but does not adopt multiple positions.

The resolution achieved allowed the modelling of α-helices and the location of haem groups and density that could be attributed to β-sheets. Moreover, there is density for the larger, aliphatic/aromatic side chains in the transmembrane region that can be modelled into the map (Figs. 2 ▸ *d*, 2 ▸ *e* and 2 ▸ *f*). At this resolution, *de novo* structure building is possible using *Rosetta*, as exemplified by the CNG channel (James *et al.*, 2017 ▸). However, given that crystal structures were already available, these were docked into the corresponding cryo-EM maps as an initial starting point, as the *de novo* building of *bc*_1_ would have been a significant undertaking. The docked crystal structures were initially subjected to flexible fitting in *MDFF* to identify any large changes at the secondary-structure level, after which *PHENIX* was used to refine the side-chain positions (Adams *et al.*, 2010 ▸). The maps were then visually inspected to look for any areas of poor fit and the residues were manually fitted, with the resulting structures being re-refined in *PHENIX*. It is important to note that as we have the phase information in the cryo-EM map, at no point in the refinement procedure was the map modified or biased by the fitted structure. Analysis of the resulting structures showed there were no significant differences between the different cryo-EM-derived structures and those solved by X-ray crystallography, except for the Rieske domain (described below).

**Figure 3:**
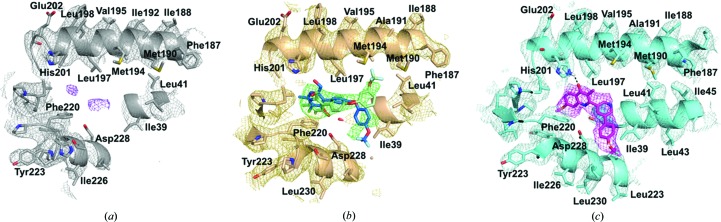
Analysis of the Q_i_ site in the three different cryo-EM maps with the density contoured at 3σ. (*a*) The Q_i_ site in the apo *bc*_1_ cryo-EM map shows minimal density (purple mesh) which does not correspond to any side chains or the haem *b*_L_ group, suggesting noise within the map or that the natural substrate ubiquinone is bound in a small number of *bc*_1_ molecules. (*b*) The Q_i_ site in the cryo-EM map of *bc*_1_–GSK932121. The inhibitor density (green) suggests there are two modes of inhibitor binding, accompanied by rotation around the oxygen–carbon bond. The binding pose shown in green agrees with the crystal structure, with the trifluoromethyl group pointing towards Met194. There is additional density which suggests that the trifluoromethoxyphenyl group could be rotated and point towards Asp228, revealing an additional mode of binding (shown in blue). (*c*) The Q_i_ site in the cryo-EM map of *bc*_1_–SCR0911, with the inhibitor shown in pink and located in strong density. The inhibitor is expected to make a hydrogen-bond contact with His201 and strongly fits the density.

The Q_i_ site in all three models has been analysed to determine whether inhibitor density can be seen (Fig. 3 ▸). In the apo structure there is weak density for the natural substrate, ubiquinone, at the Q_i_ site, which is consistent with several studies that found ubiquinone bound to the Q_i_ site (Huang *et al.*, 2005 ▸; Hao *et al.*, 2012 ▸). Owing to the lower resolution of the apo map, the side chains of the neighbouring amino-acid residues could not be unambiguously modelled into the density for all residues; however, many could be placed into their approximate positions (Figs. 2 ▸ and 3 ▸). For the two inhibitor-bound structures, there is clear inhibitor density at the Q_i_ site which does not appear at the Q_o_ site, supporting that these are selective Q_i_ site inhibitors. The strongest inhibitor density occurs in the *bc*_1_–SCR0911 map, where the density for the compound is strong and comparable to that of the neighbouring side chains (Fig. 3 ▸ *c*). The density within the Q_i_ site suggests that SCR0911 adopts a conformation which is consistent with the crystal structure. The quinolone head of SCR0911 is placed between residues His201 and Phe220, and the biaryl tail further extends into the hydrophobic region of Ile39 and Ala232. In contrast, the density in the *bc*_1_–GSK932121 map is strong for the pyridone head group but is weaker at the tail of the molecule. The density also suggests that there could be two binding poses of the compound caused by rotation around the oxygen–carbon bond (as discussed in more detail below). The Q_i_ sites of the two inhibitor-bound cryo-EM structures have been compared, showing a strong agreement in the secondary structure of the protein. At 4.1 Å resolution it is difficult to detect subtle changes in the positions of the amino-acid side chains which would result from inhibitor binding. However, gross changes in the side-chain position can be detected; for example, His201 shifts in position between the two maps (Fig. 5*a*). For SCR0911, His201 is well defined and is in a position consistent with hydrogen-bond formation with the inhibitor, whereas the density for GSK932121 suggests that there is no hydrogen-bond inter­action with His201 and the density is more poorly defined (Fig. 3 ▸). Comparisons of the Q_i_ site of apo *bc*_1_ and inhibitor-bound bovine *bc*_1_ in the cryo-EM structures shows that there is no difference in the position of the α-helices which surround the active site, consistent with the observations from crystal studies that show that no gross structural changes accompany inhibitor binding.

### 3.3. Comparison of cryo-EM and X-ray crystallographic structures   

**Figure 4:**
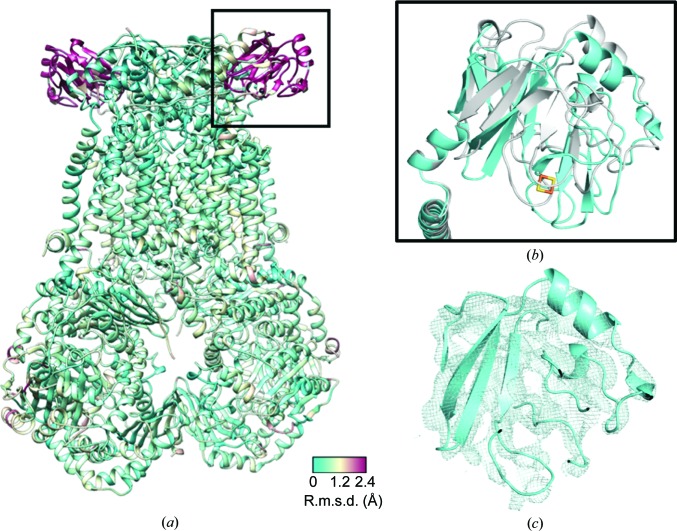
Global comparison between cryo-EM-derived and X-ray-derived SCR0911-bound structures. (*a*) The cryo-EM and X-ray *bc*_1_–SCR0911 structures have been overlaid and the crystal structure is coloured according to the calculated C^α^ r.m.s.d. value (low coloured cyan and high coloured maroon). The main difference (r.m.s.d. of >2 Å) occurs for the Rieske domain (in the black square), which is known to be mobile during the catalytic cycle. (*b*) An overlay of the Rieske domains in the cryo-EM-derived (cyan) and X-ray-derived (grey) *bc*_1_–SCR0911 structures. (*c*) The cryo-EM-derived map for the *bc*_1_–SCR0911 structure with the Rieske domain fitted shown in the same orientation as in (*a*) and (*b*).

Owing to a lack of confidence in some side-chain positions, we compared the main-chain C^α^ positions of the cryo-EM and crystal structures of the *bc*_1_–SCR0911 complex, which resulted in an r.m.s.d. of 0.5 Å. However, it should be noted that the structure, especially in poorly defined areas of the map, could still be biased by the original starting model used. There is very high agreement between the protein core and transmembrane domains. However, there are significant differences in the mobile Rieske protein in the soluble region, where the C^α^ r.m.s.d. value is greater than 2 Å (Fig. 4 ▸). This observation correlates with the crystal structures of apo, natural substrate-bound and Q_o_ inhibitor-bound bovine *bc*_1_, which capture different conformations of the Rieske protein and suggest that the swivel motion of the Rieske protein moves the 2S cluster closer to either the Q_o_ site or the cytochrome *c*_1_ haem to allow the transfer of one electron to cytochrome *c*_1_ and one electron to the Q_i_ site (Esser *et al.*, 2006 ▸). A number of different crystal structures of *bc*_1_ have shown a range of positions for the Rieske protein (Iwata *et al.*, 1998 ▸; Gao *et al.*, 2003 ▸; Esser *et al.*, 2004 ▸, 2006 ▸; Xia *et al.*, 2013 ▸; Supplementary Fig. S4). The position of this protein can be influenced by the space group in which the protein crystallizes and the corresponding crystal contacts that are made (Supplementary Fig. S4*a*), and the nature of the inhibitor bound in the Q_o_ site (Supplementary Fig. S4*b*). In the case of the cryo-EM structures we have removed the influence of the crystal lattice and therefore the Rieske domain is not being influenced by the crystal contacts, and we note that the domain does not adopt a large number of defined states, although its resolution is significantly lower than that of the main body of the protein, indicating a degree of flexibility (Fig. 4 ▸ *c*). In the cleaved N-terminal part of the Rieske protein (chain *I* residues 1–78), we can only see two β-sheets assigned as the C-terminal part of the fragment in the crystal structure.

**Figure 5:**
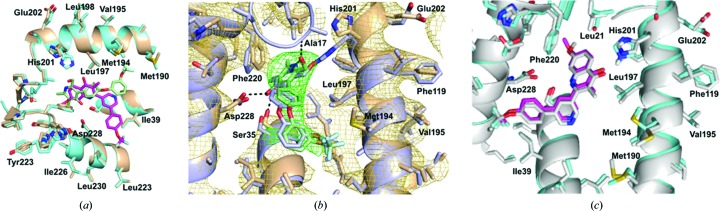
Comparisons of the Q_i_ site. (*a*) The cryo-EM structures of *bc*_1_–SCR0911 (cyan) and *bc*_1_–GSK932121 (gold) overlaid. The SCR0911 inhibitor is coloured green and the GSK932121 inhibitor magenta. The overlay of the two structures shows there is no difference in the secondary-structure positions in the two maps and only minor differences in the positions of the amino-acid residues. The largest difference occurs for His201, which forms a hydrogen bond to SCR0911 but not GSK932121. (*b*) An overlay of the Q_i_ site of *bc*_1_–GSK932121 in the cryo-EM (gold) and X-ray (purple) models. The density in gold corresponds to the EM map, which shows a difference in the position of His201 compared with the X-ray structure. (*c*) An overlay of the Q_i_ site of *bc*_1_–SCR0911 in the cryo-EM (cyan) and X-ray (grey) models. There are no significant differences between the two models in either side-chain or secondary-structure positions.

Analysis of the Q_i_ site of *bc*_1_–SCR0911 shows that the ligand adopts a very similar position in the cryo-EM-derived and X-ray-derived structures (Fig. 5 ▸). In both structures the ligand can form hydrogen bonds to His201 and Ser35 on the neighbouring helix. The Q_i_ site amino acids adopt similar positions in both structures, with an average C^α^ r.m.s.d. value of 1.46 Å; there is a clear overlap of the α-helices. There are minor differences in the positions of the amino-acid side chains, such as Phe220; however, this could be owing to the difference in the resolutions of the two maps.

Examination of the Q_i_ site in the *bc*_1_–GSK932121 cryo-EM map suggested that the inhibitor binds in two different conformations (Fig. 5 ▸ *b*). Both binding modes have the pyridone head placed between haem *b*_H_ and His201 and have the diaryl tail group extending out of the hydrophobic channel away from the haem group. The difference in the binding conformations occurs at the 4-trifluoro­methoxyphenyl ring of the molecule, as there is rotation around the C—O bond which results in two different binding positions (Fig. 3 ▸ *b*). Pyridone binding pose 1 has the trifluoromethoxy group from the second aromatic ring pointing towards Met194. In comparison, the cryo-EM structure has identified pyridone binding pose 2, which shows the aromatic ring facing in the opposite direction with the trifluoromethoxy group towards Asp228. Comparisons with the crystal structure reveal that the GSK932121 ligand adopts one binding position. As the additional binding pose was not identified using crystallo­graphy, cryo-EM could provide unique insights into alternative modes of binding and occupancy of inhibitors.

### 3.4. Comparisons of isolated *bc*_1_ with the full respirasome complex   

The structure of the porcine respiratory supercomplex (I_1_III_3_IV_1_) has been determined at 4.0 Å resolution (Wu *et al.*, 2016 ▸). After masking of the individual complexes the global resolution of the map for the *bc*_1_ structure was improved to 3.6 Å, which could be owing to the protein being stabilized by its interactions with the other components in the electron-transport chain. In the full complex, no density was present at either the Q_i_ or Q_o_ inhibitor-binding sites, which suggests that there is no natural substrate present in this map.

Our cryo-EM-derived structure of isolated *bc*_1_–SCR0911 was compared with the *bc*_1_ map which was extracted from the supercomplex cryo-EM map, with the largest differences being found in the iron–sulfur cluster (Supplementary Fig. S5). The crystal structure with the SCR0911 inhibitor bound was also compared with the full respirasome complex. Interestingly, the positions of the Rieske domains were similar when these two models were compared (Supplementary Fig. S5*b*). This could be a feature of the higher resolution of the two maps. Moreover, electron density is present in the supercomplex for subunit 11 in *bc*_1_, which is not seen in either the X-ray or cryo-EM structures of the isolated complexes owing to it being lost during the purification procedure (Supplementary Fig. S5*c*).

## 4. Discussion and outlook   

New medicines to treat malaria and other apicomplexan diseases are urgently needed to deal with the growing burden that these diseases place on many developing countries. A promising approach to deal with this problem is through the inhibition of *bc*_1_, which has been shown to be a validated target against both *Plasmodium* and *Toxoplasma*, but problems associated with drug resistance and toxicity must be addressed. To achieve this, highly potent inhibitors have been developed to target the Q_i_ site. The inhibitor-binding mode has been shown for some inhibitors by structural studies of the bovine enzyme using X-ray crystallography.

The crystal structures reported here and previously have been invaluable in providing insights into further inhibitor-design and safety-profile improvement. However, a significant limitation is the reliance upon a *bc*_1_ homologue which can be extracted in the large milligram quantities required for crystallization, with a concentration of 40 mg ml^−1^ often being required to obtain high-quality crystals. This prohibits the use of native *bc*_1_ of parasite origin for crystallography as it cannot be obtained in the high quantities required for crystallization. Subsequently, alternative methods are required to solve the structure of parasite *bc*_1_ at a resolution where inhibitor binding can be seen but where the required level of protein is significantly reduced. To this end, we provide the first single-particle cryo-EM reconstructions for bovine *bc*_1_ in its apo form and in complex with two inhibitors to show proof of principle. The resolution of the cryo-EM map shows side-chain density for bulky amino-acid residues but, without the crystal structure to act as a guide. However, it would have been a challenge to build the whole structure *de novo*. Despite this, we can unambiguously identify the inhibitor-binding site in the cryo-EM maps, and using chemical constraints such as the presence of hydrogen bonds and/or *in silico* docking it is possible to gain insights into the mode of binding. Moreover, obtaining cryo-EM structures allows the protein to be solved in its native state in the absence of any possible artefacts owing to crystal contacts.

An advantage of X-ray crystallography over cryo-EM is the resolution which is routinely achieved (often <2.5 Å), but the timescale from purified protein through crystallization to structure determination remains unpredictable. This is even more challenging for membrane proteins, with crystallization remaining one of the most unpredictable steps and often being a bottleneck for many projects. For systems such as *bc*_1_ that have been established in a laboratory, crystals can be grown within ∼5 d after protein purification ready for data collection at an advanced synchrotron crystallographic beamline, with data collection, processing and initial model fitting taking place in a single day. The current example demonstrates that cryo-EM can be conducted in a similar timeframe but without the uncertainty of obtaining diffraction-quality crystals or requiring a large amount of purified protein. After obtaining a purified bovine *bc*_1_ sample, the time taken from preparation of the cryo-grids to obtaining a three-dimensional reconstruction of the sample was less than 10 d. This timescale includes ∼72 h of data collection and a week of data processing, including the initial motion-correction and classification steps. Although the crystallization step is removed, it is important to note that grid preparation and optimization is not without its own challenges. Given the rapid development in cryo-EM, particularly in detector technology and image-processing speeds, as well as the greater number of centres with high-quality microscopes, this timescale is expected to become even shorter, making the rapid screening of complex systems *via* cryo-EM a viable alternative.

For systems where large quantities of purified protein can be obtained, X-ray crystallography is likely to remain the gold-standard approach for SBDD. For instance, in this study bovine *bc*_1_ was purified and the resulting crystals diffracted to ∼3.1 Å resolution, which allowed the mode of inhibitor binding to be established. However, by producing more selective inhibitors, which no longer have high affinity for the model system used for structural studies, alternative approaches may be required. No structural data for the *bc*_1_ complex from *P. falciparum* are available, thus hindering the current SBDD programmes, which are based upon mammalian homology models. Crystallography is not a viable route owing to the large quantities of protein that are required and the uncertainty that is imposed by crystallization. Based on the current study, we propose that cryo-EM is a viable route for studying large membrane-protein complexes from both the host and, by analogy, the parasite, and their interactions with bound inhibitors.

## Supplementary Material

PDB reference: cryo-EM structure of apo cytochrome *bc*_1_, 6fo2 (EMDB-4288)

PDB reference: cryo-EM structure of cytochrome *bc*_1_ complex with GSK932121, 6fo0 (EMDB-4286)

PDB reference: cryo-EM structure of cytochrome *bc*_1_ complex with SCR0911, 6fo6 (EMDB-4292)

PDB reference: X-ray structure of bovine cytochrome *bc*_1_ complex with SCR0911, 5okd

Supplementary Figures and Tables.. DOI: 10.1107/S2052252518001616/ua5001sup1.pdf
