# Supplementary material for: X-ray and cryo-EM structures of inhibitor-bound cytochrome bc 1 complexes for structure-based drug discovery
Source: IUCrJ. 2018 Feb 20;5(Pt 2):200–10. doi: 10.1107/S2052252518001616 (PMC5947725; doi:10.1107/S2052252518001616)
Supplement: Supplementary file 1 [file m-05-00200-sup1.pdf]

IUCrJ

Volume 5 (2018)

Supporting information for article:

X-ray and cryo-EM structures of inhibitor-bound cytochrome *bc*$_1$ complex for structure-based drug discovery

Kangsa Amporndanai, Rachel M. Johnson, Paul M. O'Neill, Colin W.G. Fishwick, Alexander H. Jamson, Shaun Rawson, Stephen P. Muench, S. Samar Hasnain and Svetlana V. Antonyuk

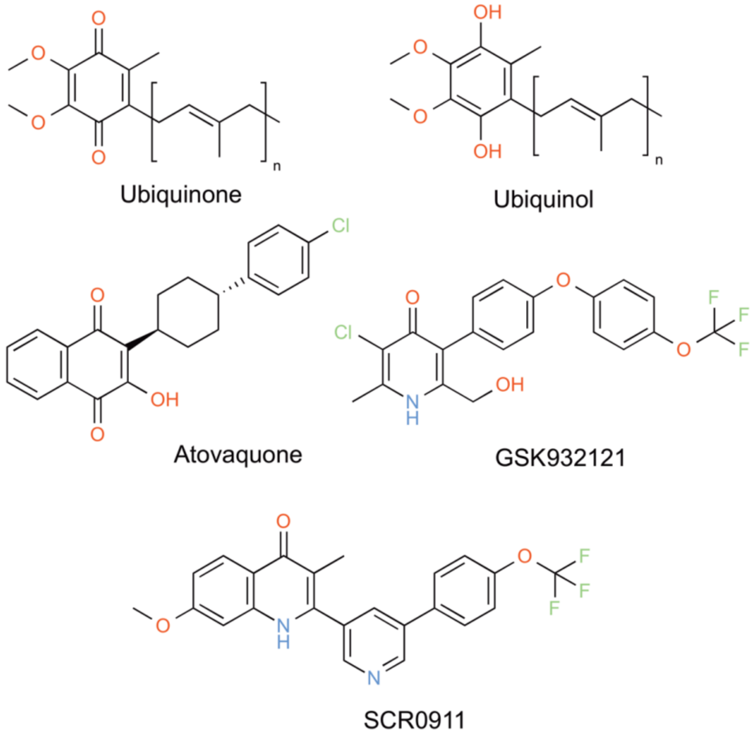

Figure S1 Chemical structures of natural substrates and selected bc$_1$ inhibitors. Ubiquinone and ubiquinol, native substrates binding to the Q$_o$ and Q$_i$ site in Q cycle. Atovaquone, anti-malarial drug inhibiting the Q$_o$ site. GSK932121, a 4(1H)-pyridone lead compound with strong antimalarial properties but failed in FTIH trials. SCR0911, 2-pyridyl-4(1H)-quinolone lead compounds developed as a Q$_i$ binder with potent inhibitory profile and improved solubility.

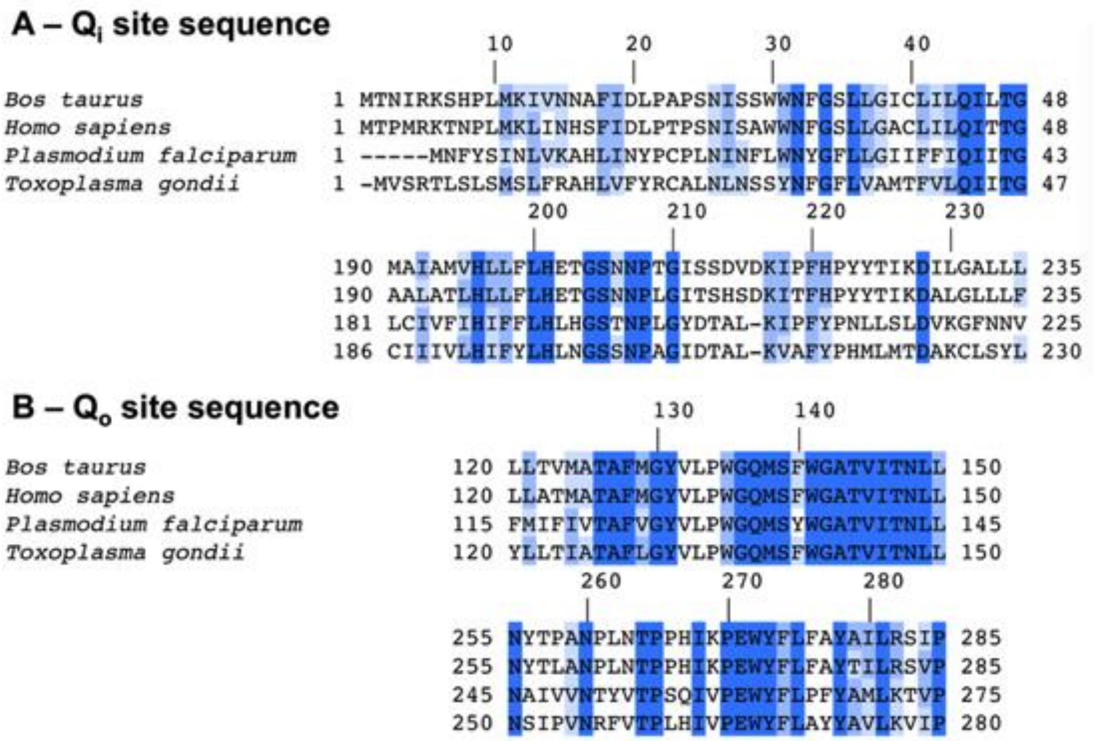

Figure S2 Protein sequence alignment between bovine, human, Plasmodium falciparum and Toxoplasma gondii. A) conservation in the Q_i site B) conservation in the Q_o site. Residues fully conserved between human and parasite are illustrated in deep blue, partially conserved in light blue, and unconserved in white. Sequence numbers at the top are shown according to the bovine sequence.

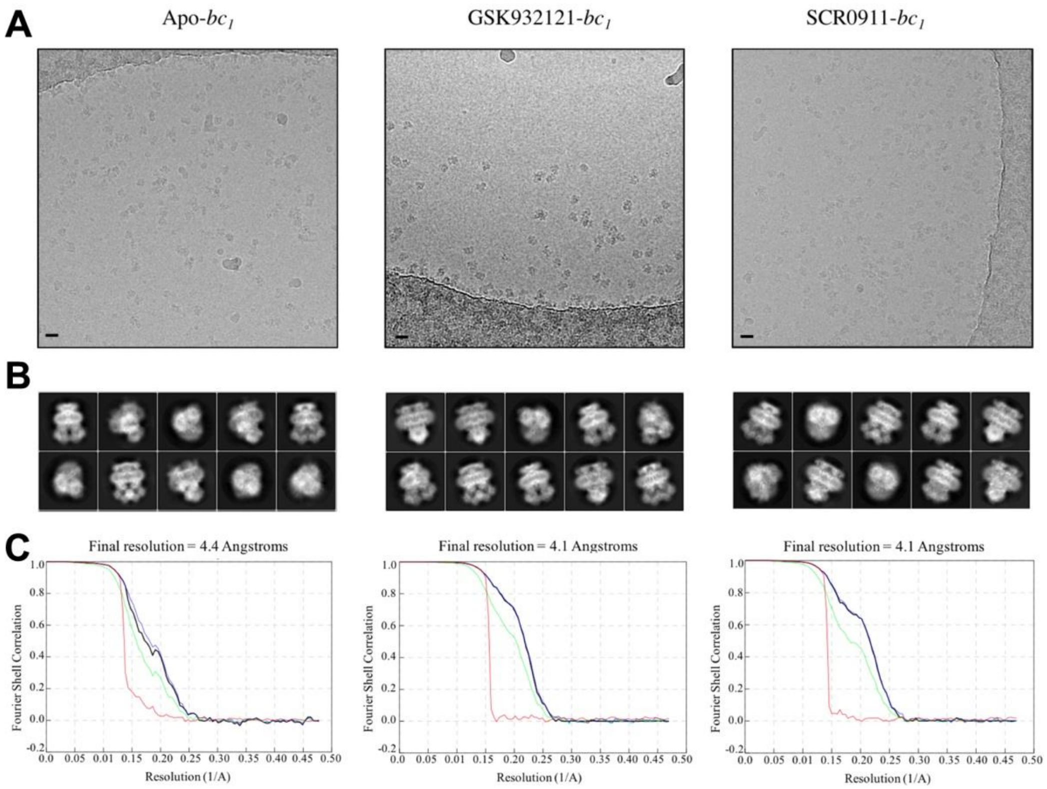

Figure S3 Summary of all of the cryo-EM data collections. A) Representative micrographs for Apo, GSK and SCR-bound bc_1 complexes which highlight a monodisperse distribution of particles in the ice for each sample (scale bar 18 nm). B) 2D classes for each data collection generated in RELION which show a wide range of orientations within the ice. Within the classes the overall shape of the protein is clearly visible, including the detergent micelle and there is enough detail within the 2D classes to see secondary structure information in particular within the transmembrane helices. C) The FSC curves for each data set to show how the resolution was calculated using the 0.143 gold standard, the curves are coloured black, green, blue and red for the corrected map, unmasked map, masked map and phase randomised map, respectively.

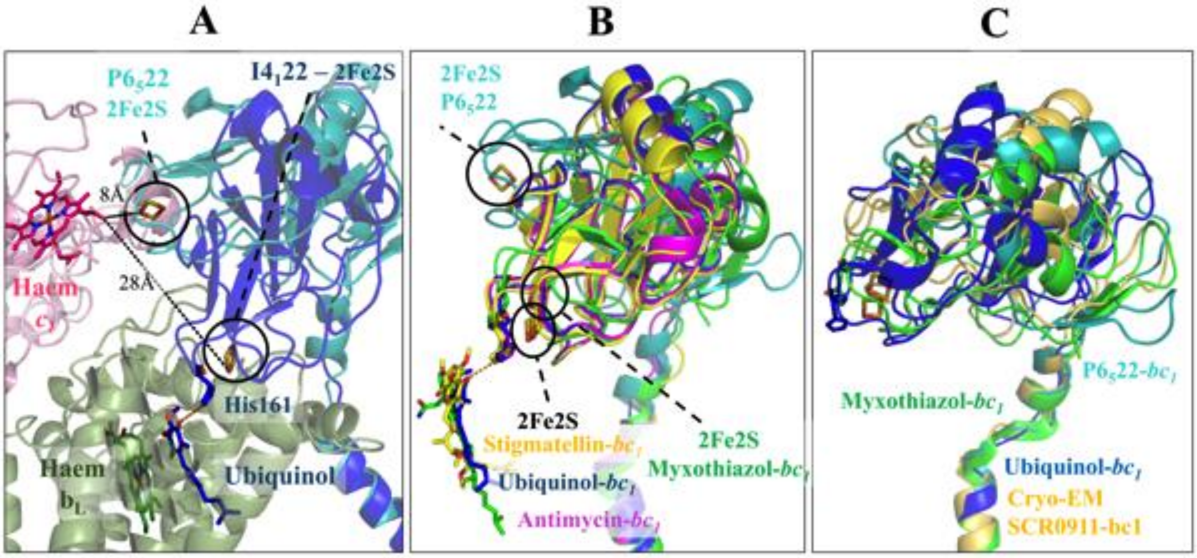

Figure S4 The superposition of the apo, native substrate, Q_o and Q_i inhibitor-bound bovine bc_1 crystal structures showing different conformations of the Rieske iron-sulphur protein. A) Cartoon representation of cytochrome b and cytochrome c_1, coloured in pink and green, respectively. Haems, ligands and amino acid residues are shown as sticks. Rieske protein from apo $P6_522$ crystal (1BE3) and ubiquinol-bound $I4_122$ crystal (1NTZ) structures are illustrated in cyan and blue, respectively. Two different space group crystals show two distinct positions of the 2Fe2S cluster. $P6_522$ crystal provides the shorter distance between Rieske protein and haem c_1, while the ubiquinol-bound $I4_122$ crystal structure induces Rieske protein closer to the Q_o site on Cytochrome b. Ubiquinol can form a H-bond (shown as orange dash line) to His161 that immobilises the Rieske protein. B) Rieske protein from apo bc_1 $P6_522$ (1BE3), ubiquinol (1NTZ), stigmatellin (1SQX), myxothiazol (1SQP) and antimycin (1NTK) bound bc_1 structures are coloured in cyan, blue, yellow, green and pink cartoon, respectively. The Q_o site bound ligands are shown as sticks. C) Cartoon representation of the Rieske protein conformation derived by cryo-EM (pale yellow) superimposed with other structures from Fig. S5B. Apo bc_1 in $P6_522$ crystal form, ubiquinol- and myxothiazol bound bc_1 in $I4_122$ crystal form are coloured in cyan, blue and green, respectively.

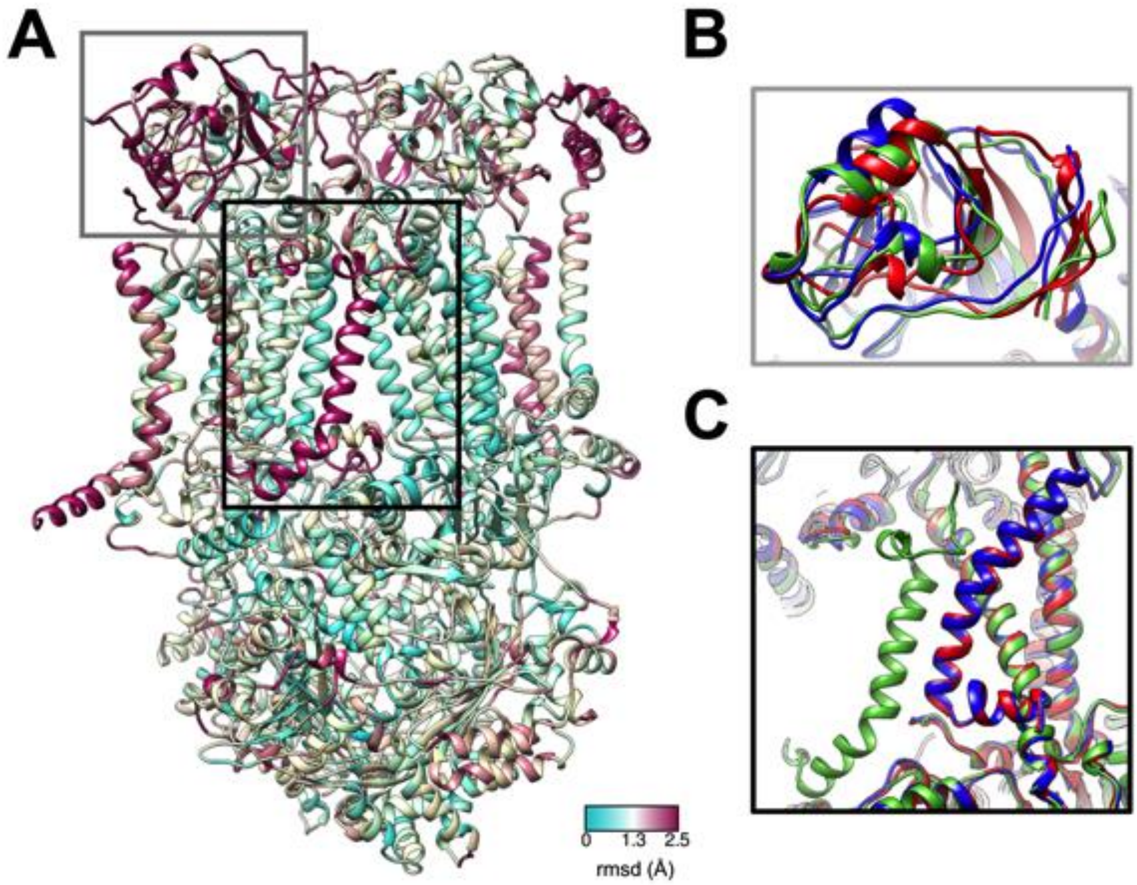

Figure S5 Comparison of the SCR0911-bc$_{1\ cryo-}$EM and SCR0911-bc$_1$ crystal structures with the bc$_1$ complex extracted from the full respiratory complex (PDB:5GUP). A) The coordinates for the bc$_{1\text{-SCR0911}}$ crystal structure and bc$_1$ complex from the full respirasome were overlaid in chimera and the C_α r.m.s.d. value was calculated. The cyan represents little difference between the two models whereas the maroon colour shows a greater shift (>2 Å). The biggest differences occur in the Rieske domain (grey square) and the hinge α-helices, which are mobile within the catalytic cycle. There is also a big difference in the position of subunit 11 (black square). B) The Rieske domain of the SCR0911-bc$_1$ X-ray (red) and cryo-EM (blue) structures overlaid with the bc$_1$ extracted from the cryo-EM supercomplex structure (green) of the full respirasome showing movement of the α-helices in this domain. C) Superposition of the three structures (coloured as in B) showing movement of subunit 11. In the full respirasome complex, this subunit is involved in interactions with the complex IV, which could explain the difference in its position.

Table S1 Bovine cytochrome bc_1 inhibition

**Compound**	**Bovine bc_1 (%inhibition at 100nM)**	**Bovine bc_1 (%inhibition at 1μM)**
GSK932121	64	81
SCR0911	9	72

***** Bovine bc_1 inhibition assay was carried out in 50mM KPi pH 7.5, 2mM EDTA, 10mM KCN, 30μM equine cytochrome *c* (Sigma-Aldrich), and 2.5 nM bovine cytochrome bc_1 at room temperature. The inhibitors were added to the assay without prior incubation. The reaction was initiated by the addition of 50μM decylubiquinol (Sigma-Aldrich). The reduced cytochrome *c* was monitored by the different absorption between 550 and 542 nm using extinction coefficient of 18.1 $mM^{-1}cm^{-1}$ in a SPECTRAmax Plus 384 UV-visible Spectrometer.

Table S2 Data collection and refinement statistics for SCR0911 bound cytochrome bc_1

Data collection	Cyt.bc_1-SCR0911
Wavelength (Å)	0.9800
Beamline	I03
Detector	Pilatus
Space group	$P6_522$
Unit-cell dimensions<br>(a,b,c) (Å)<br>(α,β,γ) (°)	 209.83, 209.83, 344.22<br>90°, 90°, 120°
Resolution (last shell) (Å)	89.44-3.10 (3.16-3.10)
Rmerge % (last shell)	11.0 (120.0)
R_{pim} (last shell) (%)	4.3 (47.7)
CC(1/2) (last shell)	0.98 (0.57)
I/σ (last shell)	12.3 (1.8)
Completeness (%)	99.5 (99.9)
Redundancy	7.4 (7.5)
Wilson B factor (Å^2)	70
No. of unique reflections	79127
Rwork/Rfree	0.206/0.249
Atoms	
Protein	15916
SCR0911	31
waters	82
Other ligands (lipids, detergents, phosphates, PEG etc.)	472
B factor (Å^2)	
Protein overall	101
Protein chains A/B/C/D/E/F/G/H/I/J	95/96/88/115/134/90/100/143/135/109
SCR0911	75.3
Other ligands	202
Waters	73
R.M.S deviations	
Bond length (Å)	0.007
Bond angles (°)	1.26
PDB code	5OKD

Table S3 Data collection and refinement statistics for Cryo-EM structures

	***bc*$_1$-apo**	***bc*$_1$-GSK932121**	***bc*$_1$-SCR0911**
Data collection:			
Detector	K2 Summit (Gatan)	FEI Falcon III (Integrating mode)	FEI Falcon III (Integrating mode)
Voltage (kV)	300	300	300
Pixel size (Å)	1.047	1.065	1.065
Defocus (μm)	-1 to -4	-1 to -4	-1 to -4
Total dose (e^-/ Å)	44	75	85
No. of frames	20	50	40
Dose per frame	2.20	1.50	2.125
No. of micrographs	3,256	8,840	7,893
Total no. of auto-picked particles	260,201	466,865	629,258
Particles in final refinement	57,571	232,910	114,130
Refinement:			
Resolution (C2)	4.4 Å	4.1 Å	4.1 Å
R.m.s.d bond lengths (Å)	0.01	0.01	0.01
R.m.s.d bond angles (°)	0.99	1.11	1.02
Validation:			
Clashscore (all atoms)	4.34	4.21	3.65
Ramachandran:			
Favoured (%)	92.40	89.77	94.83
Allowed (%)	7.60	10.18	5.17
Outliers (%)	0	0.05	0
